# Assessment of Patient Satisfaction in a Military and Public Hospital: A Comparative Study

**DOI:** 10.7759/cureus.10174

**Published:** 2020-08-31

**Authors:** Ayesha Farooq, Muhammad Azeem Khaliq, Muhammad Aftab Toor, Aminah Amjad, Wakar Khalid, Farooq Butt

**Affiliations:** 1 Internal Medicine, Combined Military Hospital, Lahore Medical College, National University of Medical Sciences, Lahore, PAK; 2 House Officer, Combined Military Hospital, Lahore Medical College, National University of Medical Sciences, Lahore, PAK; 3 Medicine, Combined Military Hospital, Lahore Medical College, National University of Medical Sciences, Lahore, PAK; 4 Surgery, University of Health Sciences, Gujranwala, PAK

**Keywords:** patient satisfaction, military, pakistan, public, health systems

## Abstract

Background: In recent times, the assessment of patient satisfaction has become an essential tool for measuring the effectiveness of healthcare delivery. However, not a lot of work has been done in Pakistan, even less so in comparing it across different hospital systems in the country. This research aims to fill that gap and be the first to compare satisfaction levels in a military and public hospital.

Objective: To assess patient satisfaction in different hospital systems of Pakistan and compare their outcomes.

Methods: A cross-sectional study was undertaken between October 2019 and April 2020 among 376 patients; 193 from Combined Military Hospital, Lahore (CMH) and 183 from Jinnah Hospital, Lahore. The questionnaire used for the study was the Short-Form Patient Satisfaction Questionnaire - 18, and convenience sampling was used to select participants. Data was entered and analysed on Statistical Product and Service Solutions (SPSS) version 21 (IBM Corp., Armonk, NY).

Results: The majority of participants were male (71.2%), entitled to free healthcare (58.4%), and employed (59.7%). It was found that CMH Lahore scored better in all seven domains of patient satisfaction (p<0.03 individually), with significant differences in six: general satisfaction, interpersonal manner, communication, financial aspects, time spent with the doctors, and accessibility and convenience. Overall, waiting times and entitlement to free healthcare were established to be major determinants of satisfaction, with CMH having shorter waiting times and providing free treatment to a larger number of patients. The mode waiting time in CMH was 1 - 15 minutes (44.9%) as compared to 15 - 30 minutes (50.9%) in Jinnah Hospital. Additionally, 78.2% of patients were entitled to free healthcare in CMH, compared to 35.5% in Jinnah Hospital.

Conclusion: Patient satisfaction was found to be significantly better in CMH in six out of seven domains studied. Further work needs to be done in its assessment, as well as in its role in healthcare policies.

## Introduction

Current literature contains many definitions for the term "patient satisfaction". However, its description as the degree to which the patients’ expectations of care have been met compared to actual care received can be considered to be one of the most comprehensive [1].

The factors which affect patient satisfaction are numerous, and can be divided into two main categories: major and minor. Major factors include waiting time, nursing care, interpersonal communication, time spent with the doctor, hospital services provided, and treatment outcome [2,3]. Minor factors are generally non-modifiable including gender, age, health, and educational status. However, their subjectivity has led to conflicting opinions on how they affect patient satisfaction [4].

There are many benefits associated with increased patient satisfaction levels, including but not limited to: increased compliance to medication, improved interpersonal relations and productivity, increased patient loyalty, enhanced market share of hospitals, and decreased malpractice claims [5-8].

The role of assessing patient satisfaction in modern healthcare has been gaining importance in recent years, being considered an index of medical service [9]. Patient satisfaction has also been described by Donabedian as an “ultimate validator of the quality of care” [10]. Additionally, the concept of patient-centred healthcare has been emerging, with patients becoming more aware of their health conditions [11]. This is strengthened by the fact that the World Health Organization (WHO) considers people-centredness to be a defining feature of patient care [12]. There is no doubt concerning the need for its assessment, which was made compulsory by Germany in 2005 [2].

The specific need for the evaluation of patient satisfaction in Pakistan is two-fold. Firstly, a gap exists in local literature pertaining to Donabedian’s third measure of patient care, i.e. outcome. The measurement of patient satisfaction is an integral component of patient outcome, as it reflects the efficacy of the process, i.e. services provided. In order to assess if and what changes are to be made to existing healthcare structures and policies, patient satisfaction must be considered. Additionally, it is important to note that Donabedian’s framework of healthcare has been modified over time. The initial model is comprised of three measures of healthcare, i.e. structure, process, and outcome. However, many alterations have been made, including its incorporation into the Integrated Chronic Disease Management (ICDM) model, which was designed for the management of noncommunicable diseases in South Africa [13]. 

The additional need for the assessment of patient satisfaction in Pakistan lies in its current healthcare system. Unlike other countries that have predominantly one system, e.g. the NHS, Pakistan’s delivery of healthcare can be considered three-fold, with the interplay of public, private, and military-based institutions.

It is important to note that military hospitals encompass army, air force, and naval institutions. Of these three, army hospitals have the strongest presence, being located in many major cities of Pakistan, as well as in regions otherwise facing socioeconomic disparity like Khuzdar, Balochistan. Army hospitals are named Combined Military Hospitals (CMH), as they also provide free-of-cost treatment to those serving in the air force and navy. Conversely, they provide non-entitled patients care at full cost, with the cost of one consultation in CMH Lahore currently at 1000rs ($6.00 USD). The current number of functional CMHs in Pakistan is 45 [14].

On the other hand, public hospitals are much more numerous, being scattered across the country. In 2016, there were a total of 1279 public hospitals operating in Pakistan [15]. They provide treatment at highly subsidised rates for all members of the population. The cost of consultation in Jinnah Hospital, Lahore is currently 50rs ($0.30 USD). For the underprivileged, there are no expenses.

Private hospitals are also scattered throughout the country, being more numerous in urban areas. There were a total of 700 private hospitals in the country in 2016 [15]. All patients are required to pay for treatment, however the amount may vary. An average cost of consultation can range from 300 - 5000rs ($1.79 - $30.02 USD).

As public, private, and military hospitals each play a significant role in the deliverance of healthcare to the population, it becomes imperative to evaluate their effectiveness at both an individual and collective level. Not only will this highlight distinctive areas of strengths and weaknesses, it will also provide suggestions to overcome perceived shortcomings based on patient feedback, thus strengthening the process.

Currently, there are gaps in local literature comparing these systems - specifically military establishments. Considering their impact on healthcare, the objective of this research is to assess, as well as compare, satisfaction levels in a public and military hospital. It is hoped that this study will not only help fill the present gap in literature, but also aid in the suggestion and implementation of policies directly affecting patient satisfaction.

## Materials and methods

This cross-sectional study was carried out from October 2019 to April 2020. A total of 376 patients took part in the study divided amongst two tertiary care hospitals in Lahore, Pakistan, namely: Combined Military Hospital (CMH) with 193 participants and Jinnah Hospital, a public institution, with 183 participants. Convenience sampling was used to select participants, who were given the opportunity to partake on a voluntary basis.

The inclusion criteria consisted of patients either waiting outside medical/surgical outpatient departments (OPDs), or admitted in wards. These included general OPDs and wards as well as allied specialties such as dermatology and urology. Patients admitted in intensive care facilities were not included, as well as those with neurocognitive impairment e.g. dementia.

The questionnaire used for data collection was divided into two segments: Section A for demographics and Section B for the assessment of satisfaction, using the short-form Patient Satisfaction Questionnaire (PSQ-18) - an internationally validated, self-administered form consisting of 18 questions. Each question requires the patient to reflect on how much they agree or disagree with a given statement, with their responses being graded on a scale of 1-5; 1 being very unsatisfied and 5 being very satisfied. The PSQ-18 assesses seven domains of satisfaction: general satisfaction, technical quality, interpersonal manner, communication, financial aspects, time spent with the doctor, and accessibility and convenience.

The questionnaire was translated to Urdu, the national language, and a pilot study was conducted in CMH consisting of 20 patients. Shortcomings were noted and the questionnaire was amended accordingly, before being distributed en masse.

Ethical approval was sought from the ethical board of both hospitals before initiating the study. Verbal consent was taken before handing out the forms which were filled out anonymously. Literate patients independently completed their forms, whereas illiterate patients were given the option of participating via an interview. A member of the research team was always present as an overseer to address any queries raised by participants.

Data was entered and analysed on Statistical Product and Service Solutions (SPSS) version 21 (IBM Corp., Armonk, NY). The mean score for each domain was calculated and the scores for both hospitals were compared with each other using the independent T test. For continuous variables such as age and waiting time, Pearson’s correlation was used to assess their relationships with satisfaction. Results were considered significant at p<0.05.

## Results

Out of the 376 patients participating in the research, 71.2% were male and 28.8% female. The majority of patients were below the age of 50 (66%), and employed (59.7%). More than half of subjects were married (67.2%) and had completed their education above 10th grade (57.5%). Patients largely belonged to the province of Punjab (63.7%), followed by Khyber Pakhtunkhwa (15.1%) and Balochistan (13.2%). Demographic data is displayed in Table 1.

**Table 1:** Patient Profile and Demographics

CHARACTERISTIC		N	%
Age (yrs.)	18 - 34	123	33.2
	35 - 49	122	32.9
	50 - 64	81	21.8
	≥ 65	41	11.1
Gender	Male	265	71.2
	Female	107	28.8
Hospital	Combined Military Hospital	193	51.3
	Public	183	48.7
Entitlement Status	Yes	216	58.4
	No	152	41.1
Employment Status	Yes	221	59.7
	No	149	40.3
Marital Status	Married	250	67.2
	Single	122	32.8
Educational Status	Intermediate and above	214	57.5
	Matric or below	156	41.9
Province	Punjab	237	63.7
	Khyber Pakhtunkhwa	56	15.1
	Baluchistan	49	13.2
	Gilgit Baltistan	14	3.8
	Kashmir	9	2.4
	Sindh	7	1.9

The collective scores for mean satisfaction levels in each domain, in descending order, were as follows: accessibility and convenience (3.67±0.80), communication (3.64±0.88), financial aspects (3.59±0.95), technical quality (3.40±0.74), general satisfaction (3.36±0.82), time spent with the doctor (3.23±0.98), and interpersonal manner (3.16±0.91). These values have been elucidated in Table 2.

**Table 2:** Mean Values for Domains of Satisfaction Assessed by the Patient Satisfaction Questionnaire-18 (PSQ-18)

Domain of Satisfaction	Minimum	Maximum	Mean	Standard Deviation
General Satisfaction	1.0	5.0	3.363	.8249
Technical Quality	1.3	5.0	3.401	.7389
Interpersonal Manner	1.0	5.0	3.169	.9114
Communication	1.0	5.0	3.638	.8821
Financial Aspect	1.0	5.0	3.590	.9472
Time Spent with the Doctor	1.0	5.0	3.225	.9767
Accessibility and Convenience	1.0	5.0	3.669	.8018

**Figure 1:**
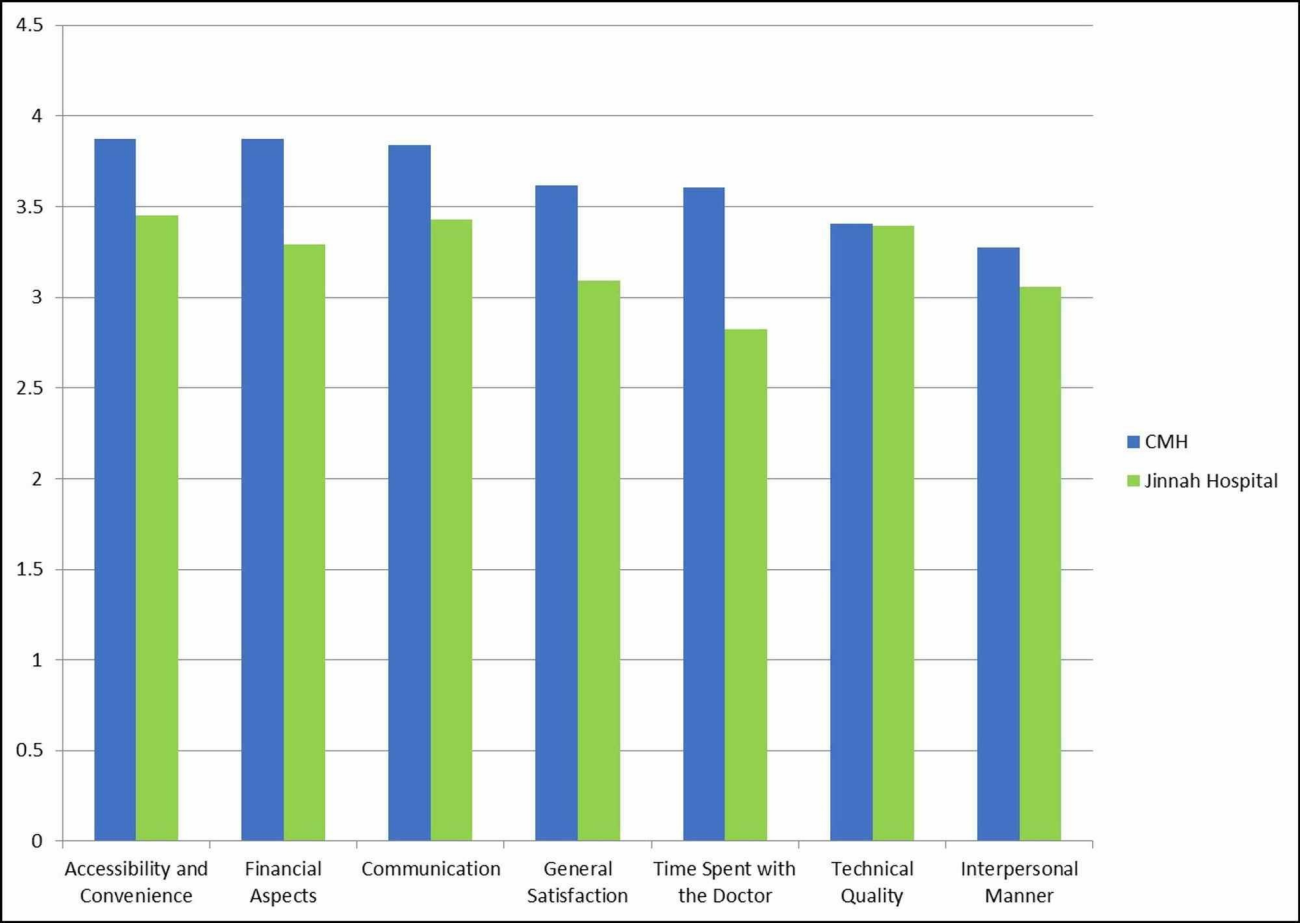
Comparison of Patient Satisfaction in CMH and Jinnah Hospital CMH = Combined Military Hospital

In relation to subject figures, approximately equal numbers of patients belonged to each hospital type; 193 (51.3%) questionnaires were recorded in CMH whilst 183 (48.7%) in Jinnah Hospital. Patients reporting to CMH were most satisfied with accessibility and convenience (3.88±0.85), followed by financial aspects (3.87±0.92), communication (3.84±0.95), general satisfaction (3.62±0.89), time spent with the doctor (3.60±0.99), technical quality (3.40±0.86), and interpersonal manner (3.27±0.99). Meanwhile, patients reporting to Jinnah Hospital were most satisfied with accessibility and convenience (3.45±0.69), followed by communication (3.43±0.75), technical quality (3.40±0.60), financial aspects (3.29±0.88), general satisfaction (3.09±0.65), interpersonal manner (3.06±0.81), and time spent with the doctor (2.83±0.78). This is displayed in Figure 1. Furthermore, it was found that satisfaction levels between hospitals was also statistically significant. Patients in CMH were significantly more satisfied in terms of accessibility and convenience, financial aspects, communication, general satisfaction, time spent with the doctor, and interpersonal manner (p<0.03 each).

**Figure 2:**
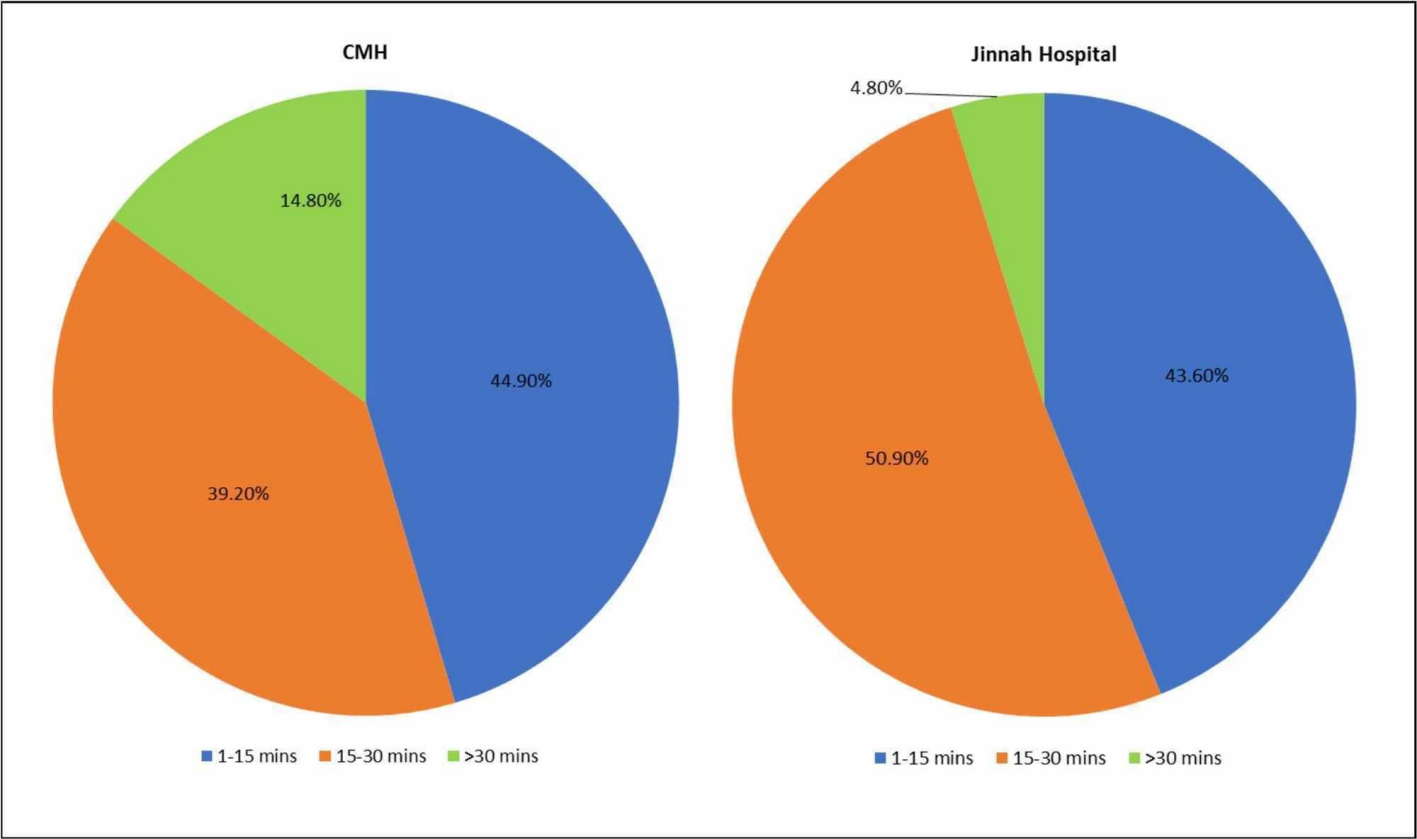
Comparison of Waiting Times in CMH and Jinnah Hospital CMH = Combined Military Hospital

Disparities amongst waiting time were also found, with patients in CMH waiting less than those in Jinnah Hospital for their consultations, as seen in Figure 2. The collective modes for waiting time were: 1 - 15 minutes (44.3%), 15 - 30 minutes (44.9%) and greater than 30 minutes (10.8%). In addition, it was found that patients in CMH recorded 1 - 15 minutes their mode waiting time (44.9%), whereas patients in Jinnah Hospital recorded 15 - 30 minutes (50.9%). Linear trends were also found with waiting time and satisfaction. As waiting times increased, patients recorded decrease in satisfaction levels in terms of general satisfaction (p=0.008), communication (p=0.029), financial aspect (p=0.001), and accessibility and convenience (p=0.005).

**Figure 3:**
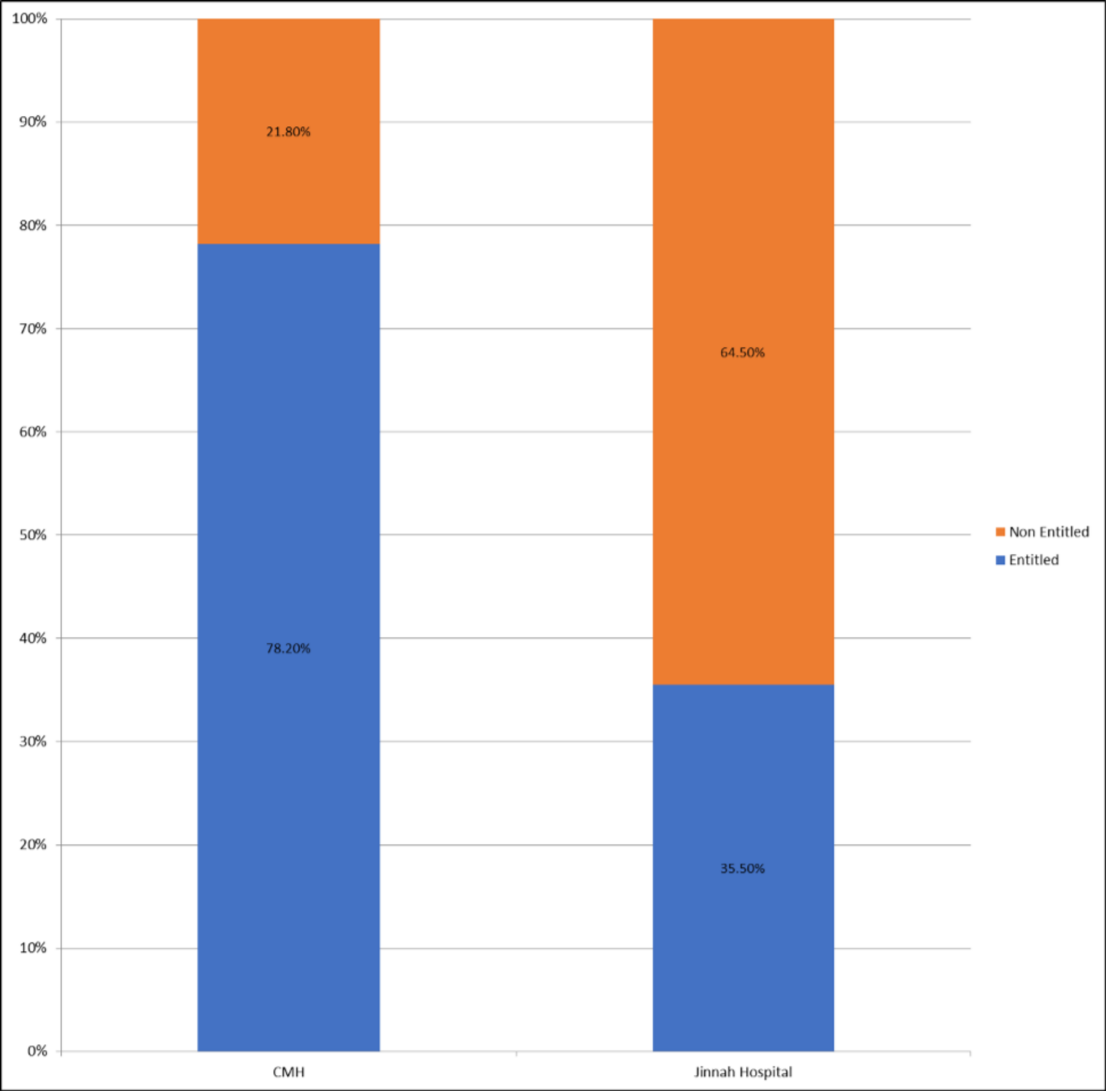
Proportion of Patients Entitled to Free Healthcare in CMH and Jinnah Hospital CMH = Combined Military Hospital

A total of 216 patients (58.4%) were cumulatively entitled to free treatment, of which 151 (69.9%) belonged to CMH. This is further elaborated in Figure 3. Entitled patients as a whole were most satisfied with communication and least satisfied with interpersonal manner. On the other hand, paying patients were most satisfied with accessibility and convenience while least satisfied with time spent with the doctor. Furthermore, it was found that patients who were eligible for free care were more satisfied than those who were not, in terms of general satisfaction (p=0.021), communication (p=0.000), financial aspect (p=0.000), time spent with the doctor (p=0.000), and accessibility and convenience (p=0.024).

Employment status impacted satisfaction levels as well. Employed persons were most satisfied with the financial aspects, while least satisfied with interpersonal manner. In contrast, unemployed persons were most satisfied with accessibility and convenience, while least satisfied with time spent with the doctor. Satisfaction levels between the two groups were also comparable - employed persons being significantly more satisfied in financial aspects (p=0.004), and time spent with the doctor (p=0.002).

It was found that more patients reported from medicine and allied departments (63%) as opposed to surgery and allied (37%). As a whole, patients were most satisfied with accessibility and convenience, whilst least satisfied with technical quality (medicine and allied) and time spent with the doctor (surgery and allied). However, no significant difference was recorded between their satisfaction levels.

Non-modifiable factors such as gender and age were also seen to influence satisfaction levels. Males were most satisfied with accessibility and convenience, whereas females preferred communication. Both parties were least satisfied with interpersonal manner. In terms of comparative statistics, males were significantly more satisfied than their female counterparts with regards to technical quality (p=0.044), financial aspects (p=0.007), and accessibility and convenience (p=0.003). Moreover, age was seen to negatively correlate with satisfaction, with increasing age associated with decreased satisfaction in time spent with the doctor (p=0.002).

## Discussion

Key findings

The objective of this study was to bring to light the salient differences in satisfaction experienced by patients attending military and public hospitals. CMH scored better in all seven domains of satisfaction assessed by the PSQ-18; moreover, these differences were found to have statistical significance in general satisfaction, interpersonal manner, communication, financial aspects, time spent with the doctor, and accessibility and convenience. Individually, time spent with the doctor and interpersonal manner were domains that scored poorly in both hospitals, highlighting their need for assessment. On the other hand, both sets of patients were most satisfied with accessibility and convenience.

In the evaluation of factors affecting satisfaction, waiting time and eligibility for free healthcare were determined to be major factors, with increased waiting time and lack of entitlement leading to decreased satisfaction levels. However, while comparing hospitals, not only did CMH have lesser waiting times, it also provided free healthcare to a larger number of patients (as part of amenities provided to serving personnel in the armed forces). 

Minor factors such as employment status also affected satisfaction, with increased levels seen in patients with an occupation. Satisfaction levels between employed and unemployed persons were also statistically significant in terms of financial aspects. Other factors such as age and gender were seen to impact satisfaction levels, but to a lesser extent.

Comparison with existing literature

Patients in our research were found to be overall most satisfied with accessibility and convenience, while least satisfied with interpersonal manner. Previous researches carried out in Pakistan show both similar and dissimilar results. Research carried out in Azad Kashmir similarly found patients being most satisfied with accessibility and convenience, while another study conducted in Islamabad found patients to be most satisfied with interpersonal manner. However, time spent with the doctor unanimously scored poorly, indicating its need to be addressed [16-17].

CMH scored better than Jinnah Hospital in all domains of satisfaction assessed by the PSQ-18. When comparing previous literature evaluating differences in public and private healthcare, it was found that patients attending private hospitals were overall more satisfied than those attending public institutions at a national level [17-18]. On the other hand, international results varied. Lesser developed countries like Ghana with similar health care structures as Pakistan showed comparable results to our study [19], while countries with better established and funded healthcare systems such as the United Arab Emirates declared insignificant differences between satisfaction levels [20].

In line with our research, other studies also determined that increased waiting times negatively impacted patient satisfaction [21-22]. A research carried out in CMH Malir Cantonment, Karachi also found that patients were not satisfied with waiting time, linking it to increased doctor-patient ratios. Additionally, they assessed waiting times outside pharmacies and diagnostic facilities, which were both found to be dissatisfactory [23].

Results for employment status are also comparable, with increased satisfaction being seen in employed persons [24]. The interplay of affordability, medical cost, and insurance has been extensively discussed in the literature. Comparable to our study which showed that patients entitled to free medical care were more satisfied than others, one research showed increased affordability positively affecting satisfaction levels, while high medical bills were a chief cause of dissatisfaction [25]. As expected, medical insurance also plays a role in patient satisfaction. While the concept of individually acquiring health insurance is not very popular in Pakistan, the government launched the Prime Minister National Health Program (PMNHP) in 2017, whose aim was to provide free of cost medical treatment to those living under the poverty line [26]. Local research conducted to assess satisfaction levels of patients utilising the service has shown positive results [27]. International studies also support the notion that health insurance significantly increases satisfaction [19,24,28].

While our research was able to determine influences of non-modifiable factors such as gender and age on satisfaction levels, previous literature has shown these associations to be inconsistent, and should not be relied upon while considering hospital management [4]. One of the primary reasons for this is the subjective nature of these factors and the consideration of personal preferences and values which can be difficult to assess.

Strengths and weaknesses

To our knowledge, this research is the first of its kind to be carried out in Pakistan, comparing patient satisfaction between military and government healthcare systems. To date, there has only been one study done in Pakistan assessing patient satisfaction in a military hospital [23], while comparisons have been made between public and private healthcare systems. As a whole, very little research has been done in Pakistan pertaining to patient satisfaction as it is not considered a major factor in management programs or policy making.

Our study was conducted using an internationally validated questionnaire, the Short-Form Patient Satisfaction Questionnaire (PSQ-18). This questionnaire was translated into Urdu, allowing the local population to understand and independently fill their forms. Moreover, patients who were illiterate were given the option to give oral responses, which were documented by a team member. Initially, a pilot study was done with 20 questionnaires in order to identify and address any shortcomings. To make things easier for the participants, a team member was always present to cater to any queries. 

Lastly, this research highlights major factors causing satisfaction and dissatisfaction amongst patients, thus allowing hospitals management programs and healthcare workers to effectively address their inadequacies. 

A major limitation of this research was the fact that data was obtained from one military and one public hospital only. As there might be differences in satisfaction in other setups, it may be difficult to generalize our results to the whole country.

Another important limitation in our literature was that all comparisons in our research were made using studies comparing public and private hospitals, since there was no readily available data containing military hospitals. CMH cannot truly be likened to a private hospital as the majority of patients are not required to pay fees. Thus, the strength of our comparisons is questionable.

In terms of participants, there was underrepresentation of many subgroups within the hospitals. Firstly, due to low literacy rates in our country many illiterate patients were unable to participate, as only a limited number could be interviewed. Moreover, patients who were critically ill or were admitted with cognitive impairment could not participate. However, for patients who did participate, the level of honesty could not be judged. This is particularly true for patients in CMH as the majority were military personnel who were hesitant in criticising their establishment.

Finally, it was difficult to assess patient satisfaction in its true essence. While satisfaction is a subjective feeling, our study did not include questions about the desires and expectations of our participants. Therefore, it was not possible to assess the impact of different values on satisfaction levels. Similarly, the effect of health status on satisfaction was also not studied. Since there were no questions inquiring about the health status of participants, its impact on satisfaction could not be ascertained.

Implications

The implications for this research are multiple. In terms of hospital management and policy, this study can help healthcare workers better understand the factors influencing patient satisfaction, including ways to improve its delivery.

Major factors having detrimental effects on satisfaction were deemed to be time spent with the doctor and waiting time. Changes in hospital policy can help address these salient problems, including the introduction of appointments. The current system operating in Pakistan includes patients attending OPDs on their designated days - however, the number of patients attending these clinics are not controlled. The introduction of appointments can help curb these issues as the effect is three-fold: firstly, patients will have decreased waiting time as their appointments are scheduled. Secondly, as there will be a limited number of patients in waiting and a standard time allotted for appointments, clinicians will be able to spend more time with their patients. Thirdly, the doctor-patient ratio will improve, which will all, in turn, increase patient satisfaction.

Nevertheless, in order to implement these changes, it is suggested that better funding be provided to the health care system. In 2017 Pakistan spent only 2.90% of its GDP on healthcare, in contrast to the United States which spent 17.06% [29]. Public institutions need to have better funding in order to decrease the gap between public and private hospitals, as well as between public and military. (It is important to note here that due to lack of available data, the proportion of military spending on healthcare could not be discerned.)

At an individual level, it is suggested that not only doctors, but all healthcare workers attend courses/workshops where they are introduced to the concept of patient satisfaction. As this is an area largely undiscussed in Pakistan’s health system, it is important for health workers to be made aware of its importance, as well as the benefits associated with it, such as increased compliance to medication and improved interpersonal relations.

**Figure 4:**
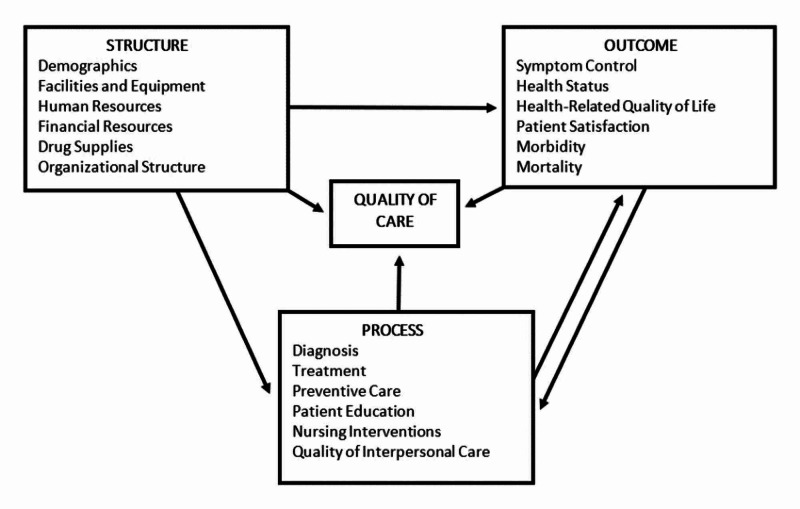
Donabedian’s Framework of Quality of Care in the Modified ICDM Model ICDM = Integrated Chronic Disease Management

Further research could be carried out by increasing the number of hospitals sampled. As our research compared one public and one military hospital, it would be interesting to carry out this comparison across multiple cities. Not only would this make the results more reliable, but would also allow them to be generalised across the population. Moreover, questions pertaining to patient expectations and education, as well as health status and nursing care received could be added to the questionnaire in order to have a more comprehensive approach to the assessment of satisfaction, in accordance to the ICDM model, which has been elaborated below in Figure 4. It is based off the ICDM model designed in South Africa [13], as previously mentioned.

Additionally, as the structure of CMH also caters to civil patients at full fees, this subset can be comparable to patients attending private hospitals. Further research could be carried out to assess these groups and identify similarities/differences. This would allow both systems to identify paths of improvement by comparison.

Lastly, this study could be repeated in the same setting after a number of years. This would enable us to understand trends in satisfaction and how they have improved/worsened over time, while at the same time assessing changes made to health care policies.

## Conclusions

As a whole, patients attending CMH were generally more satisfied than those in Jinnah Hospital. Patient waiting times were found to be shorter, while a greater number of patients were entitled to free healthcare. Furthermore, the importance of patient satisfaction in patient management cannot be over-emphasized, especially in regions where it is not given its due importance. Further assessment in Pakistan is required at multiple levels, individually as well as in comparison to other hospital systems. It is suggested that its impact be understood by all healthcare workers, and methods of improvement be employed, for example improving interpersonal manners at a singular level, and decreasing cost of healthcare at a national level.

## Appendices

Assessment of Patient Satisfaction in a Military and Public Hospital: A Comparative Study

All information you provide here is strictly confidential

Section 1: Demographics

Tick an option for each of the following:

1.     Age:   18-34☐      35-49☐       50-64             ☐≥65

2.     Gender:   Male☐           Female☐

3.     Hospital:   CMH☐          Public☐

4.     Department:   Medicine☐       Surgery☐

5.     Employed:   Yes☐          No☐

6.     Married:   Yes☐   No☐

7.     Province: Punjab☐        Balochistan☐        KPK☐         Sindh☐        Gilgit Baltistan☐          Kashmir☐

8.     Education: Intermediate or above☐   Matric or below☐

9.     Are you getting free treatment:   Yes☐       No☐

10.   How long do you have to wait before the doctor sees you:   1 - 15mins☐          15 - 30min☐             > 30min☐

*Section 2: Patient Satisfaction Questionnaire - 18 *  [30]

The next set of questions pertain to your feelings/attitudes towards the medical care you have received at your hospital. Please read each question carefully and answer as honestly as you can.

**Table 3:** Short-Form Patient Satisfaction Questionnaire - 18 (PSQ-18)

No.	Question	Strongly agree	Agree	Uncertain	Disagree	Strongly disagree
1	The medical care I have been receiving is completely satisfactory	1	2	3	4	5
2	I am dissatisfied with some aspects of the medical treatment I receive	1	2	3	4	5
3	I think my doctor’s office has everything needed to provide complete medical care	1	2	3	4	5
4	Sometimes doctors make me wonder if their diagnosis is correct	1	2	3	4	5
5	When I go for medical care, everything is carefully checked and examined	1	2	3	4	5
6	I have some doubts about the ability of the doctors who treat me	1	2	3	4	5
7	Doctors act too impersonal towards me	1	2	3	4	5
8	My doctors treat me in a very friendly and courteous manner	1	2	3	4	5
9	The doctor describes the reasons for the medical tests adequately	1	2	3	4	5
10	Doctors sometimes ignore what I tell them about my health and sickness	1	2	3	4	5
11	I feel confident I can afford the medical care I need	1	2	3	4	5
12	I have to pay more for my medical care than I can afford	1	2	3	4	5
13	Doctors here hurry too much when they treat me	1	2	3	4	5
14	I am completely satisfied with the amount of time doctors spend with me	1	2	3	4	5
15	I have easy access to the medical specialists I need	1	2	3	4	5
16	People have to wait too long for emergency treatment here	1	2	3	4	5
17	I find it hard to get a doctor’s appointment I need right away	1	2	3	4	5
18	I am able to get medical care whenever I need it	1	2	3	4	5

Thank you for completing this questionnaire.
